# Quercetin Suppresses CYR61-Mediated Multidrug Resistance in Human Gastric Adenocarcinoma AGS Cells

**DOI:** 10.3390/molecules23020209

**Published:** 2018-01-24

**Authors:** Ho Bong Hyun, Jeong Yong Moon, Somi Kim Cho

**Affiliations:** 1 Faculty of Biotechnology, College of Applied Life Sciences, SARI, Jeju National University, Jeju 63243, Korea; fhdighqhd@nate.com; 2 Subtropical/Tropical Organism Gene Bank, Jeju National University, Jeju 63243, Korea; owenmjy@jejunu.ac.kr; 3 Faculty of Advanced Convergence Technology & Science, Jeju National University, Jeju 63243, Korea

**Keywords:** apoptosis, CYR61, multidrug resistance (MDR), quercetin, synergistic effect

## Abstract

Cysteine-rich angiogenic inducer 61 (CYR61) is an extracellular matrix-associated protein involved in survival, tumorigenesis, and drug resistance. Therefore, we examined the effects of flavones against CYR61-overexpressing human gastric adenocarcinoma AGS (AGS-cyr61) cells, which show remarkable resistance to 5-fluorouracil (5-FU), adriamycin (ADR), tamoxifen (TAM), paclitaxel (PAC), and docetaxel (DOC). Among the tested flavones, quercetin had the lowest 50% inhibitory concentration (IC_50_) and significantly reduced the viability of AGS-cyr61 cells compared with AGS cells. Quercetin: (1) reduced multidrug resistance-associated protein 1 and nuclear factor (NF)-kappa B p65 subunit levels; (2) reversed multidrug resistance (MDR); (3) inhibited colony formation and induced caspase-dependent apoptosis; and (4) suppressed migration and down-regulated epithelial–mesenchymal transition-related proteins in AGS-cyr61. Moreover, AGS-cyr61 cells treated with quercetin concentrations close to the IC_50_ and simultaneously treated with 5-FU or ADR in the sub-lethal range showed strong synergism between quercetin and these two drugs. These findings indicate that CYR61 is a potential regulator of drug resistance and that quercetin may be a novel agent for improving the efficacy of anticancer drugs in AGS-cyr61 cells.

## 1. Introduction

Gastric cancer is a major public health problem globally and the second leading cause of death from malignant tumors, with a five-year survival rate of only about 20% [1]. Many lines of evidence suggest that overexpression of the CCN family (cysteine-rich protein [CYR61], connective tissue growth factor [CTGF], and nephroblastoma overexpressed gene [Nov]) increase migration, peritoneal dissemination, and carcinogenic progress in gastric cancer cells [2,3]. Notably, CYR61 was highly expressed in a more advanced gastric adenocarcinoma specimen, and the human gastric cancer cell line overexpressing CYR61 significantly increased tumor cell motility and invasion through activation of the integrin/nuclear factor-kappa B (NF-κB) p65/cyclooxygenase-2 signaling pathway. Increased CYR61 levels have been reported to induce increased proliferation and invasion [4], and induce resistance to apoptosis and paclitaxel (PAC) in breast cancer cells [5,6]. Although CYR61 has been implicated in chemoresistance of breast cancer cells, no previous studies have investigated the relationship between CYR61 and drug resistance in gastric cancer. Because of the importance of chemotherapy in gastric cancer treatment, multidrug resistance (MDR) is a major obstacle to effective chemotherapy. Therefore, it is necessary to study the relationship between CYR61 and drug resistance to identify CYR61-targeting agents in stomach cancer [7]. CCN-targeting agents, including monoclonal antibodies, antisense oligonucleotides, and RNA interference compounds, have been proposed [8]. However, in the long term, the safety of this trial strategy and its economy have not been clinically proven. Furthermore, there is a need to identify CYR61-targeting agents because there are limited reports on agents that can target CYR61 signaling.

Flavonoids are a group of naturally occurring low-molecular-weight polyphenol compounds that have recently become the subject of therapeutic interest based on considerable scientific evidence. Flavonoids are found in various plants and are present in fruits, roots, stems, and flowers, and are consumed in the form of daily meals, tea, and wine [9]. The flavone 2-phenyl-4*H*-1-benzopyran-4-one contains phenyl rings substituted with hydroxyl or methoxyl groups. Importantly, hydroxyl groups can increase solubility, whereas methoxyl groups increase cell permeability [10]. The presence of methoxyl groups on the polyphenolic backbone (*O*-methylated flavones) has been suggested to lead to superior chemopreventive properties to the more common unmethylated flavonoids or polyphenols [11], and the number of methoxyl groups influence the activity [12]. The most well-known flavones, such as nobiletin (5,6,7,8,3′,4′-hexamethoxyflavone), tangeretin (5,6,7,8,4′-pentamethoxyflavone), and quercetin (3,5,7,3′,4′-pentahydroxyflavone), induce apoptosis of cancer cells and sensitization, improving the efficacy of drugs [12]. Moreover, these flavones potentiate the growth inhibition of several chemotherapeutic agents, such as 5-FU, PAC, and cisplatin, through different intracellular mechanisms in different types of cancer cells [13,14,15]. However, the detailed mechanism of the reversion of drug resistance by these flavones in gastric cancer remains unknown.

The molecular genetic basis of resistance to cancer therapeutics is complex because it involves several processes, including drug delivery, drug metabolism, DNA repair, and apoptosis [16]. For example, ATP-binding cassette (ABC) transporters are a group of transporter proteins that contribute to drug resistance through ATP-dependent drug efflux pumps. The ABC transporter family has at least 48 members in humans, 12 of which are thought to be putative drug transporters, including P-glycoprotein, multidrug resistance protein 1 (MDR1; encoded by the *ABCB1* gene), and MDR-associated protein 1 (MRP1; encoded by the *ABCC1* gene) [17]. Some ABC transporters confer chemoresistance by causing an efflux of anti-cancer drugs [18]. Although the mechanisms of MDR are complex [19], overexpression of P-glycoprotein in tumor cells is the one of the main causes of MDR [20]. Considering the low cost, proven safety, and pharmacological efficacy of flavones, we examined their anticancer activity against CYR61-overexpressing human gastric adenocarcinoma AGS (AGS-cyr61) cells to identify flavones that can target CYR61. We demonstrated that quercetin is an effective agent that targets CYR61 in gastric cancer cells and downregulates NF-κB p65 and MRP1. In addition, we investigated the chemo-adjuvant action of quercetin in combination with the standard chemotherapeutic agents 5-fluorouracil (5-FU) and adriamycin (ADR). This study provides evidence of quercetin as a novel agent that inhibits MDR and enhances drug sensitivity in CYR61-overexpressed gastric cancer patients.

## 2. Results

### 2.1. Multidrug Resistance in AGS-cyr61 Cells

**Figure 1:**
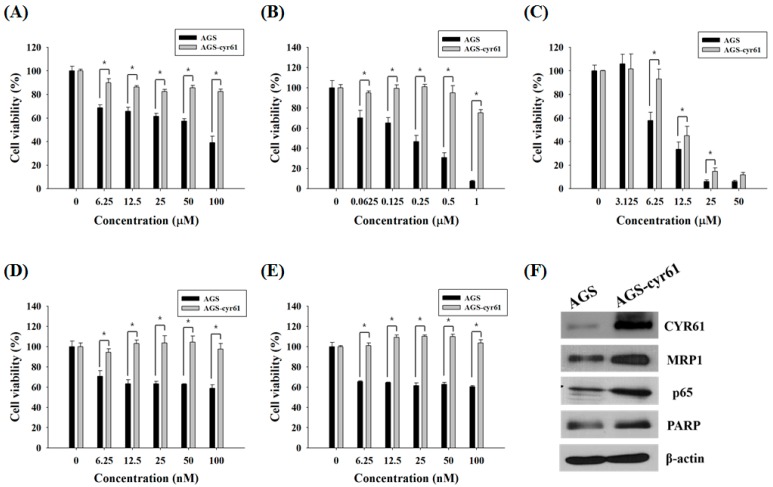
Cell growth inhibition by anticancer drugs and characterization of AGS-cyr61 cells. Cell viability was determined based on the 3-(4,5-dimethylthiazol-2-yl)-2,5-diphenyl-tetrazolium bromide (MTT) reduction assay against AGS and AGS-cyr61 cells treated with (**A**) 5-FU; (**B**) ADR; (**C**) TAM; (**D**) PAC; and (**E**) DOC for 48 h. Values are the means ± standard deviation (SD) of four independent experiments. * *p* < 0.05 compared to AGS and AGS-cyr61 cells. (**F**) Cysteine-rich angiogenic inducer 61 (CYR61), multidrug resistance (MDR)-associated protein 1 (MRP1), p65, and poly (ADP-ribose) polymerase (PARP) proteins in AGS and AGS-cyr61 cells were analyzed by western blot. β-Actin was used as an internal control.

Because it has been reported that CYR61 is associated with PAC and ADR resistance in breast cancer cells [21], we first examined whether CYR61 is related to MDR in gastric cancer cells. AGS cells treated with 5-FU, ADR, or TAM showed dose-dependent declines in cell viability, although the dose-response curves of PAC and DOC plateaued at 12.5 nM and 6.25 nM, respectively. Meanwhile, AGS-cyr61 cells exhibited notable resistance to 5-FU, ADR, PAC, and DOC, and slight resistance to TAM (Figure 1A–E). The 50% inhibitory concentration (IC_50_) values of 5-FU and ADR in AGS-cyr61 cells (>100 μM and >1 μM, respectively) were higher than those in AGS cells (67.1 ± 1.9 μM and 0.4 ± 0.1 μM, respectively). These results indicate that CYR61 overexpression confers MDR in AGS cells, and that AGS-cyr61 cells can acquire resistance to cell death induced by 5-FU or ADR. To clarify the role of CYR61 in resistance in AGS gastric cancer cells, western blot analysis of drug resistance-related proteins was performed in both cell lines. The AGS cell lines overexpressing CYR61 showed upregulation of MRP1, NF-kB p65 subunit, and PARP (Figure 1F). These results indicate that CYR61 overexpression confers drug resistance in AGS-cyr61 via the upregulation of the drug resistance-related proteins MRP1, p65, and PARP.

### 2.2. Quercetin Downregulates Drug Resistance-Related Proteins in AGS-cyr61 Cells 

**Figure 2:**
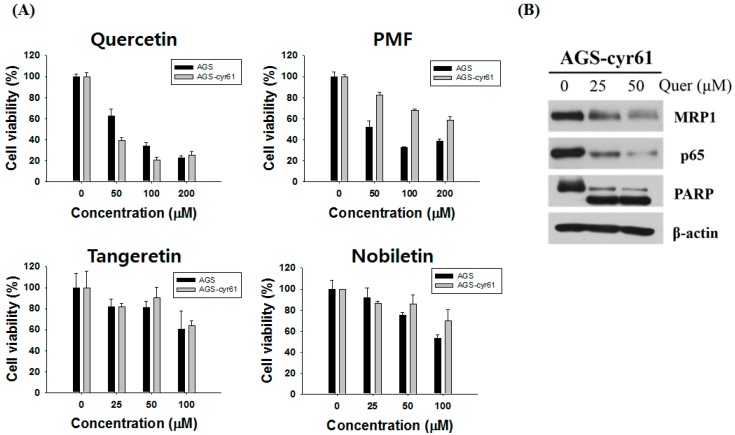
Effect of quercetin on the expression of drug resistance-related proteins in AGS-cyr61 cells. (**A**) Cell viability was determined based on the MTT assay against various flavones in AGS and AGS-cyr61 cells. Values are the means ± SD of four independent experiments. PMF: pentamethoxyflavone; (**B**) Western blot analysis of the effect of quercetin on drug resistance-related protein expression in AGS-cyr61 cells

Next, we assessed flavones for their ability to target CYR61. Since flavones have been reported to have various activities depending on their methylation and number of methoxyl groups [12], we investigated the cytotoxic effects of four representative flavones (quercetin, tangeretin, pentamethoxyflavone, and nobiletin, Table 1) with different numbers of methoxyl groups in AGS-cyr61 cells (Figure 2A). 

Among the tested flavones, only quercetin reduced the viability of AGS-cyr61 cells more than AGS cells (1.5 fold), and quercetin had the lowest IC_50_ (46.51 ± 15.17 μM) among the tested flavones (Table 1). Moreover, quercetin treatment significantly decreased CYR61, MRP1, and p65 levels and induced PARP cleavage in AGS-cyr61 cells (Figure 2B). These results indicate that quercetin has the potential to overcome MDR caused by CYR61 overexpression.

### 2.3. Quercetin Induces Apoptosis and Inhibits Colony Formation in AGS-cyr61 Cells

**Figure 3:**
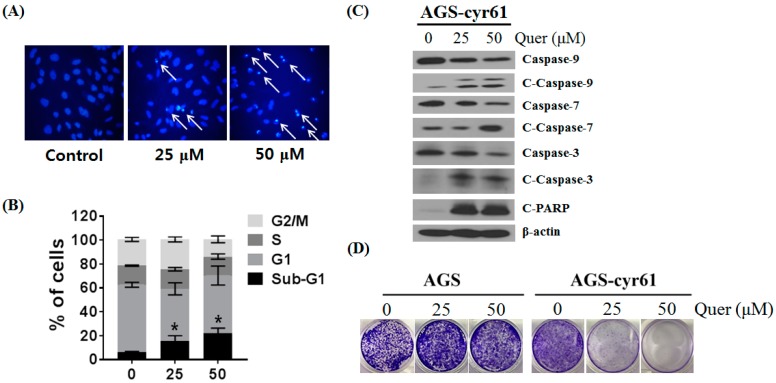
Quercetin induces apoptosis and inhibits colony formation in AGS-cyr61 cells. (**A**) Cell apoptosis observed using Hoechst 33342 staining. AGS-cyr61 cells were treated with quercetin (0, 25, and 50 µM) for 24 h. Apoptotic cells exhibited nuclear morphological changes typical of apoptosis. The stained nuclei were visualized under a fluorescence microscope. Arrows represent apoptotic cells; (**B**) AGS-cyr61 cells were treated with quercetin (0, 25, and 50 μM) and fixed after 24 h for flow cytometry. Propidium iodide-labeled nuclei were analyzed for DNA content. The sub-G0/G1 apoptotic population and the G1, S, and G2/M phase populations were quantified using DNA histograms. The data shown are representative of three independent experiments that gave similar results. * *p* < 0.05 compared with the AGS-cyr61 cell control; (**C**) Quercetin induces apoptosis via a caspase-dependent apoptosis pathway in AGS-cyr61 cells. AGS-cyr61 cells were treated with quercetin for 24 h; (**D**) Colony formation ability assay with quercetin for 5 days in AGS and AGS-cyr61 cells. AGS and AGS-cyr61 cells were stained using crystal violet for 30 min.

To investigate whether quercetin-induced AGS-cyr61 cell death was caused by apoptosis, we performed Hoechst 33342 staining, cell cycle analysis, western blotting, and a clonogenic assay. After treatment with quercetin, AGS-cyr61 cells showed obvious morphological changes (Figure 3A), including condensed chromatin and apoptotic bodies (arrows). In addition, cell cycle flow cytometric analysis showed that quercetin induced a dose-dependent increase in the sub-G1 population from 6.29 ± 0.58% (0 μM) to 21.98 ± 4.47% (50 μM) (Figure 3B). Moreover, caspase-9, -7, and -3 levels were reduced and the levels of cleaved caspase-9, -7, -3 and PARP were increased (Figure 3C). Quercetin greatly inhibited colony formation of AGS-cyr61, but no significant inhibition was detected in AGS cells (Figure 3D). These results indicate that quercetin has the potential to reverse drug resistance through the induction of apoptosis and inhibition of colony formation in AGS-cyr61 cells.

### 2.4. Quercetin Inhibits Cell Migration and Downregulates the Epithelial–Mesenchymal Transition (EMT) in AGS-cyr61 Cells

A number of studies have shown that CYR61 functions as an integrin ligand and the human gastric cancer cell line overexpressing CYR61 significantly increased tumor cell motility and invasion has pivotal roles in cell migration [16,22]. The epithelial-mesenchymal transition (EMT) is the process by which epithelial cells become mesenchymal stem cells, during which the epithelial cells lose cell polarity and cell-cell adhesion, and become mobility and invasiveness. EMT is an essential process in embryonic development, tissue remodeling and wound healing, but most importantly, it plays a crucial role in tumor invasion and metastasis [23].

**Figure 4:**
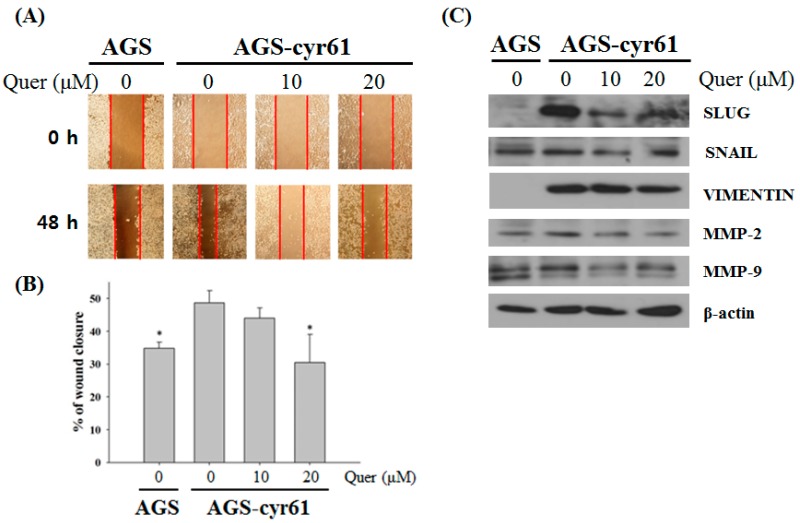
Quercetin inhibits cell migration and downregulates epithelial–mesenchymal transition (EMT)-related protein expression in AGS-cyr61 cells. (**A**) Wound-healing assay of AGS and AGS-cyr61 cells. After a confluent monolayer was formed, the cell monolayer was scratched with the sterile tip of a 200 μL micropipette. AGS cells were treated with a vehicle control (dimethyl sulfoxide [DMSO]), and AGS-cyr61 cells were treated with a vehicle control (DMSO) and quercetin (10 and 20 μM) for 48 h; (**B**) Percentage wound closure 48 h after DMSO and quercetin treatment, as in (**A**). Data are the means ± standard error for three independent experiments. * *p* < 0.05 compared with the control of AGS-cyr61 cells; (**C**) AGS-cyr61 cells were treated with quercetin for 24 h. Cell lysates were analyzed by western blotting for EMT-related proteins.

Moreover, a link between the epithelial–mesenchymal transition and cancer cell drug resistance has been suggested [24]. Therefore, the effects of quercetin on AGS-cyr61 cell mobility was examined by calculating the percentage wound closure by comparing the distance of remaining cell-free area to the distance of initial cell-free area. After the test, control untreated AGS-cyr61 cells covered most of the wound area, which was significantly larger than that of control AGS cells. The control AGS-cyr61 cells, which were treated with DMSO vehicle only, covered most wound areas during the 48-h incubation period, with a percentage wound closure of 48.67 ± 3.77% (Figure 4A,B), which was significantly larger than that of the control AGS cells. When AGS-cyr61 cells were exposed to 20 μM of quercetin, closure was reduced to 30.51 ± 8.61%. Next, we detected the expression of epithelial and mesenchymal phenotype marker proteins using western blotting. Compared with AGS cells, AGS-cyr61 cells exhibited increased levels of EMT-related proteins, including slug, snail, vimentin, matrix metalloproteinase (MMP)-2, and MMP-9. Consistent with the wound healing assay results, mesenchymal marker, slug, snail, and vimentin protein levels were reduced in quercetin-treated AGS-cyr61 cells (Figure 4C). These results indicate that quercetin inhibits AGS-cyr61 cell migration by inhibiting EMT-related protein expression.

### 2.5. Quercetin Synergizes the Actions of the Standard Chemotherapeutic Agents 5-FU and ADR

**Figure 5:**
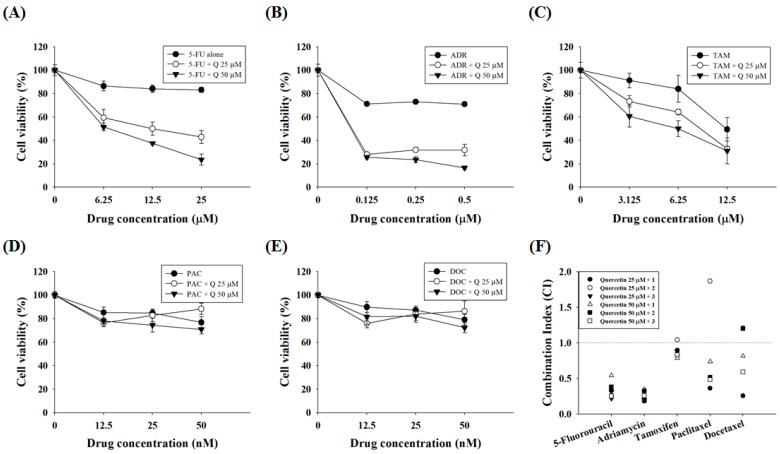
Quercetin synergizes the actions of standard chemotherapeutic drugs in AGS-cyr61 cells. AGS-cyr61 cells were treated with 25 and 50 µM quercetin (Q) and the standard chemotherapeutic drugs (**A**) 5-FU; (**B**) ADR; (**C**) TAM; (**D**) PAC; and (**E**) DOC in AGS-cyr61 cells for 48 h. The MTT assay was used to measure cell viability; (**F**) Combination index (CI) value was calculated using the program CalcuSyn. CI values of <1, 1, and >1 were considered to indicate synergistic, additive, and antagonistic effects, respectively.

Because quercetin shows combination effects with some DNA-damaging drugs in colorectal and prostate cancer cells [25,26], the synergistic effects of quercetin with standard chemotherapeutic agents were assessed. AGS-cyr61 cells were incubated with quercetin and subtoxic doses (~90% cell viability in AGS-cyr61 cells relative to the control) of standard chemotherapeutic agents, and cell viability was determined using the MTT assay. In AGS-cyr61 cells treated with 50 μM quercetin + 25 μM 5-FU (23.50% of cell viability), cell viability decreased more than 59% compared with cells treated with 5-FU alone (83.19%) (Figure 5A). Treatment with 50 μM quercetin + 0.5 μM ADR inhibited cell growth more than 54% compared with cells treated with ADR alone (71.02%) (Figure 5B). The combination index (CI)-value method provides a quantitative calculation of synergism between drugs. A CI is assessed from dose-effect data of single and combined drug treatments. A value of CI less than 1 indicates synergism; CI = 1 indicates additive effect; and CI > 1 indicates antagonism. Drug interaction (synergism or antagonism) is more distinct the farther a CI value is from 1. The combination index (CI) was determined using CalcuSyn software (Biosoft, Cambridge, UK) and expressed as the average of the CI values obtained for three different combinations. The findings showed that 25 and 50 μM quercetin and sub-lethal doses of 5-FU had a strong synergistic effect (CI = 0.21–0.54, Table 2). In addition, quercetin showed a synergistic effect with ADR (CI = 0.18–0.34, Table 2). Meanwhile, TAM showed a weak synergistic effect (CI = 0.78–0.89), except for 25 μM of quercetin and 6.25 μM of TAM (CI = 1.03), whereas PAC and DOC showed synergistic effects only at specific concentration ratios with quercetin (Figure 5C, Table 2).

**Figure 6:**
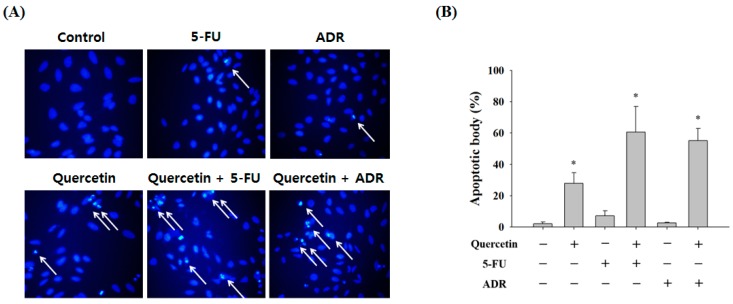
Effect of combining quercetin with 5-FU or ADR on the induction of apoptosis in AGS-cyr61 cells. (**A**) Synergistic effects of quercetin and 5-FU and ADR on apoptosis in AGS-cyr61 cells for 24 h. AGS-cyr61 cells were treated with 25 µM quercetin, 25 µM 5-FU, and 125 nM ADR. Treatment was given as one drug alone or as a combination of drugs. Apoptotic bodies were determined using Hoechst 33342 staining, and stained cells were visualized under a fluorescence microscope; (**B**) Number of apoptotic bodies. Data are the means ± standard error for one experiment performed in triplicate. * *p* < 0.05 compared with the control.

Next, we confirmed the synergistic effects of quercetin with 5-FU and ADR by Hoechst 33342 staining. A synergistic response was observed based on a marked increase in apoptotic body formation (Figure 6A). Treatment with quercetin alone resulted in 27.87 ± 6.72% apoptotic body formation, and 5-FU alone resulted in only 7.16 ± 3.25% apoptotic body formation, but co-treatment resulted in 60.59 ± 16.41% apoptotic body formation. Similarly, ADR significantly increased apoptotic body formation when the AGS-cyr61 cells were co-treated with quercetin (55.17 ± 7.76%) compared with ADR alone (2.65 ± 0.41%) (Figure 6B). These results suggest that quercetin has selective synergistic effects with anticancer agents known to cause DNA damage, such as 5-FU and ADR.

## 3. Discussion

CYR61 is well known for its roles in migration, growth, and metastasis [22,27,28]. It has been reported that CYR61 is associated with drug resistance [21,29]. However, the mechanisms of drug resistance conferred by CYR61 have not been fully described. In this study, we transfected human gastric cancer AGS cells with the *cyr61* gene to elucidate the mechanism of CYR61-mediated drug resistance. CYR61-overexpressed AGS cells showed upregulation of MRP1, p65, and PARP expression, and these cells exhibited resistance to chemotherapeutic agents such as 5-FU, ADR, TAM, PAC, and DOC.

Although the development of MDR in cancer chemotherapy is a major obstacle in the effective treatment of gastric cancer, the mechanism of MDR remains unclear. One of the most-studied mechanisms of MDR is the high expression of ABC transporters. The ABC transporter family includes, MDR1, MRP1, and breast cancer resistance protein (BCRP; encoded by the ABCG2 gene) [17]. Some ABC transporters confer chemo-resistance by causing the efflux of anti-cancer drugs [18]. MDR1 and BCRP expression levels did not differ between the two cell types (data not shown), but MRP1 protein expression was significantly higher in AGS-cyr61 cells compared with AGS cells. These results suggest that CYR61 may be implicated in MDR through the upregulation of MRP1 in gastric cancer, and that the effect of quercetin on drug resistance in AGS-cyr61 cells may be due to MRP1 protein expression inhibition. MRP1 is a well-characterized drug efflux pump belonging to the ABC transporter superfamily that can release many other chemotherapeutic agents, which can lead to MDR. MRP1 overexpression confers resistance to various anticancer drugs, such as doxorubicin, topotecan, and vincristine [30]. We speculate that resistance to ADR, 5-FU, and TAM in AGS-cyr61 cells may be related to the high levels of MRP1 protein, because these anticancer drugs showed synergistic effects when cells were co-treated with quercetin, which could inhibit MRP1 expression. However, PAC and DOC resistance may not be directly related to MRP1 expression, and it has been reported that PAC and DOC could be effluxed by ABCC10 and ABCB1 [26]. For this reason, concurrent treatment with quercetin did not overcome resistance to PAC and DOC in AGS-cyr61 cells. 

PARP is an important eukaryotic stress sensor that reacts to DNA single-strand breaks, the most common type of genomic damage, in seconds. Single-strand breaks are the most common form of DNA damage, arising directly from oxidative damage and from intermediates of other DNA repair pathways [31]. Some cancer cells display low sensitivities to available chemotherapies and develop drug resistance partly via hyper-activation of some DNA repair functions [32]. PARP inhibitors have been developed to enhance the cytotoxic effect of certain chemotherapeutic agents and are currently being studied in parallel with chemotherapy in a variety of cancer types. PARP expression was elevated in CYR61-overexpressed AGS cells (Figure 1F); therefore, we assume that this is one of the mechanisms of MDR in AGS-cyr61 cells. Interestingly, quercetin caused PARP cleavage in AGS-cyr61 cells, which can induce apoptosis (Figure 2B). The role of NF-κB has been exploited extensively in many cancer cells [33,34,35,36,37,38], and its activation confers resistance to chemotherapeutic agents. In breast cancer cells, NF-κB has been linked to resistance [39] and CYR61-induced resistance via p65-dependent XIAP upregulation in MCF-7 breast cancer cells [21]. NF-κB p65 expression was elevated in CYR61-overexpressed AGS cells (Figure 1F), indicating that an autocrine loop between CYR61 and NF-κB maintains drug resistance in AGS-cyr61 cells. Furthermore, quercetin appears to contribute to overcoming drug resistance by inducing a reduction in NF-κB p65 levels in AGS-cyr61 cells (Figure 2B).

In addition, quercetin markedly increased apoptotic bodies, the sub-G1 population, cleaved PARP, and cleaved caspase-9, -7, and -3 in AGS-cyr61 cells, which did not respond to anticancer drugs such as 5-FU or ADR. The results indicate that quercetin induces cell death in drug-resistant gastric cancer cells caused by CYR61 overexpression and that quercetin can enhance the cytotoxicity of anticancer drugs, showing strong synergistic effects with 5-FU and ADR. However, clinical use of quercetin has been limited in spite of the various physiological activities of quercetin. Because quercetin is a phytochemical with properties such as low water solubility [40], gastrointestinal instability, minimal oral bioavailability (less than 17% in rats and less than 2% in humans), and extensive first-pass metabolism to reach systemic circulation. To overcome these shortcomings, recent studies have relied on nanotechnology to develop various quercetin-loaded nanocarriers such as nanoparticles, polymeric micelles, conjugates, inclusion complexes, and nanosuspensions [41,42]. Altogether, our results provide a strong rationale for the ongoing study of quercetin in combination with drugs in cancer patients and suggest that combined administration of quercetin and other anticancer drugs can greatly improve the treatment of a wide range of therapy-refractory human tumors that exhibit CYR61 overexpression.

## 4. Materials and Methods

### 4.1. Reagents

RPMI 1640 medium, Dulbecco’s modified Eagle medium, trypsin/EDTA, and fetal bovine serum (FBS) were purchased from Invitrogen (Carlsbad, CA, USA). 3-(4,5-Dimethylthiazol-2-yl)-2,5-diphenyl-tetrazolium bromide (MTT), 5-FU, ADR, tamoxifen (TAM), PAC, and quercetin were purchased from Sigma Chemical Co. (St. Louis, MO, USA). Docetaxel (DOC) was purchased from Dong-A Pharmaceutical (Seoul, Korea). MRP1, p65, poly (ADP-ribose) polymerase (PARP), β-actin, horseradish peroxidase (HRP)-conjugated goat anti-rabbit IgG (H + L), and HRP-conjugated goat anti-mouse IgG antibodies were purchased from Cell Signaling Technology (Danvers, MA, USA). CYR61 antibody was purchased from Santa Cruz Biotechnology (Dallas, TX, USA).

### 4.2. Cell Culture

The AGS cell line was obtained from the Korean Cell Line Bank (Seoul, Korea). AGS and AGS-cyr61 cells were cultured in RPMI 1640 medium supplemented with 10% heat-inactivated FBS and 1% antibiotics at 37 °C in a humidified atmosphere containing 5% carbon dioxide.

### 4.3. Gene Transfection

The expression vector CYR61 was constructed by placing human CYR61 cDNA in the pcDNA3.1 eukaryotic expression vector containing the neomycin gene under the control of the same promoter. The CYR61-sense expression constructs were transfected into AGS cells using Lipofectamine 2000 (Invitrogen). At 24 h after transfection, the cells were serum-starved for 16 h and lysed for transient transfection analysis. After G418 selection, we isolated a single clone, AGS-Cyr61.

### 4.4. Measurement of Cell Viability

Cell viability was determined using the MTT assay, which involves the conversion of MTT into formazan crystals via mitochondrial dehydrogenases. Briefly, cells were plated in 96-well plates at an initial density of 2 × 10^4^ cells/mL per well. After incubation for 24 h, the cells were treated with various concentrations of the tested compounds. At the indicated time points, 20 μL of MTT solution (5 mg/mL) was added to each well and the cells were placed in a humidified environment for 3–4 h. The supernatant was removed and dissolved in 150 μL of dimethyl sulfoxide (DMSO). Absorbance was detected using a microplate reader at 570 nm (Tecan, Salzburg, Austria).

### 4.5. Western Blotting

After treatment, cells were collected and washed twice with cold phosphate-buffered saline (PBS). Then, the cells were lysed in lysis buffer (50 mM Tris-HCl, pH 7.5, 150 mM NaCl, 1% Nonidet P-40, 2 mM EDTA, 1 mM EGTA, 1 mM NaVO_3_, 10 mM NaF, 1 mM DTT, 1 mM PMSF, 25 μg/mL aprotinin, and 25 μg/mL leupeptin) and kept on ice for 30 min. The lysate was centrifuged at 13,000 rpm at 4 °C for 30 min, and the supernatant was stored at −70 °C until use. The protein concentration was determined using the bicinchoninic acid protein assay kit (Pierce, Rockford, IL, USA). Aliquots of lysate were separated using 7.5–15% sodium dodecyl sulfate-polyacrylamide gel electrophoresis and transferred onto a polyvinylidene difluoride (PVDF) membrane (Merck Millipore, Darmstadt, Germany) using a glycine transfer buffer (192 mM glycine, 25 mM Tris-HCl, pH 8.8, and 20% methanol (*v*/*v*)). After blocking with 5% nonfat dried milk, the membrane was incubated for 2 h with primary antibodies followed by 30 min with secondary antibodies in milk containing Tris-buffered saline and 0.1% Tween 20. All primary antibodies were used at a 1:1000 dilution, and HRP-conjugated goat anti-rabbit IgG (H + L) and HRP-conjugated goat anti-mouse IgG were used as secondary antibodies at a 1:5000 dilution. Then, the PVDF membrane was exposed to X-ray film (AGFA, Mortsel, Belgium), and protein bands were detected using a BS ECL-Plus Kit (Biosesang, Gyeonggi-do, Korea).

### 4.6. Microscopic Observation of Nuclear Morphology

Cells placed in 60-mm dishes at a concentration of 2 × 10^4^ cells/mL were treated with the tested reagents. After 24 h, 10 μM of Hoechst 33342, a DNA-specific fluorescent dye, was added to the solution in each well, and the dishes were incubated for 20 min at 37 °C. The stained cells were observed under an fluorescence microscope (Olympus, Tokyo, Japan).

### 4.7. Wound-Healing Assays

Cells were seeded in 12-well plates at a concentration of 1 × 10^5^ cells per well with growth medium. After a confluent monolayer was formed, a single scratch wound was created using the sterile tip of a 200 μL micropipette. Cells were washed with PBS to remove detached cells and cell debris. Then, each well was supplemented with growth medium containing vehicle control (DMSO) or quercetin (10 or 20 μM). Images were captured with a phase microscope at 0 and 48 h post-wounding and sample treatment to assess cell migration. The percentage of wound closure was calculated as follows:

(1) $$\mathrm{Percentage}\;\mathrm{wound}\;\mathrm{closure}\;\left(\%\right)=\frac{\left(0\;h\;\mathrm{cell}\;\mathrm{free}\;\mathrm{area}\right)-\left(48\;h\;\mathrm{cell}\;\mathrm{free}\;\mathrm{area}\right)}{0\;h\;\mathrm{cell}\;\mathrm{free}\;\mathrm{area}}\times 100$$

### 4.8. Clonogenic Assay

Cells were seeded in 12-well plates at a concentration of 5 × 10^3^ per well in growth medium. After 1 day, cells were treated with quercetin and anticancer drugs and monitored. Colonies were fixed with 500 μL of 4% paraformaldehyde per well for 30 min, stained with crystal violet (0.5% *w*/*v*) for 30 min, and washed with PBS.

### 4.9. Statistical Analysis

Data are expressed as the means ± standard deviation of three independent determinations. Significant differences between groups were determined using a one-way analysis of variance (ANOVA).

## Figures and Tables

**Table 1:**
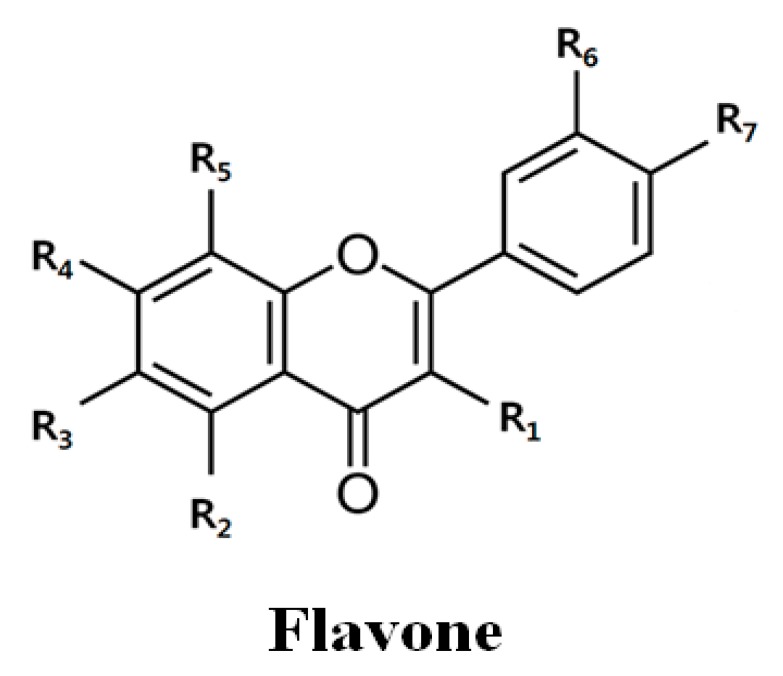
Chemical structure and IC_50_ value of flavones.

Flavones	R_1_	R_2_	R_3_	R_4_	R_5_	R_6_	R_7_	IC_50_ *	
								AGS	AGS-cyr61
Quercetin	OH	OH	H	OH	H	OH	OH	73.1 ± 9.1	46.5 ± 15.1
Tangeretin	H	OCH_3_	OCH_3_	OCH_3_	OCH_3_	H	OCH_3_	>100	>100
PMF	OCH_3_	OCH_3_	H	OCH_3_	H	OCH_3_	OCH_3_	49.8 ± 6.4	>200
Nobiletin	H	OCH_3_	OCH_3_	OCH_3_	OCH_3_	OCH_3_	OCH_3_	>100	>100

* IC_50_: 50% inhibitory concentration. PMF: pentamethoxyflavone.

**Table 2:** Combination index value of quercetin with drugs.

Quercetin	25 μM			50 µM		
Drugs	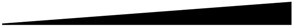			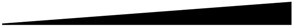		
	1 *	2	3	1	2	3
5-FU	0.33	0.26	0.21	0.54	0.38	0.25
ADR	0.18	0.20	0.20	0.34	0.32	0.25
TAM	0.89	1.04	0.81	0.78	0.83	0.83
PAC	0.36	1.86	6.87	0.73	0.51	0.48
DOC	0.25	1.20	2.96	0.81	1.20	0.59

* Different concentration of drugs: 5-FU: 6.25 (1), 12.5 (2), and 25 (3) µM; ADR: 0.125 (1), 0.25 (2), and 0.5 (3) µM; TAM: 3.125 (1), 6.25 (2), and 12.5 (3) µM; and PAC and DOC: 12.5 (1), 25 (2), and 50 (3) nM. CI values of <1, 1, and >1 were considered to indicate synergistic, additive, and antagonistic effects, respectively.
